# Nodal vulnerability to targeted attacks in power grids

**DOI:** 10.1007/s41109-018-0089-9

**Published:** 2018-08-23

**Authors:** Hale Cetinay, Karel Devriendt, Piet Van Mieghem

**Affiliations:** Delft University of Technology, Faculty of Electrical Engineering, Mathematics and Computer Science, Delft, P.O Box 5031 The Netherlands

**Keywords:** Power grids, Complex networks, Centrality metrics, Targeted attacks

## Abstract

Due to the open data policies, nowadays, some countries have their power grid data available online. This may bring a new concern to the power grid operators in terms of malicious threats. In this paper, we assess the vulnerability of power grids to targeted attacks based on network science. By employing two graph models for power grids as simple and weighted graphs, we first calculate the centrality metrics of each node in a power grid. Subsequently, we formulate different node-attack strategies based on those centrality metrics, and empirically analyse the impact of targeted attacks on the structural and the operational performance of power grids. We demonstrate our methodology in the high-voltage transmission networks of 5 European countries and in commonly used IEEE test power grids.

## Introduction

The unavailability of electrical power can severely disrupt daily life and result in substantial economic and social costs (Baldick et al. 2008). This vital importance encourages a robust design and operation of power grids (Alvarado and Oren 2002). Robust power grids are able to anticipate, adapt to and/or rapidly recover from a disruptive event or a failure.

In current practice, flow-based simulations play an essential role in the security analysis of power grids. Given the generation and demand profiles, the steady-state analyses estimate the operation of power grids. Additionally, many countries require that the power grids should withstand the scheduled and unscheduled outages of its most critical lines or other components. In these contingency analyses, the component outages are also simulated to determine whether the power grids can still function properly under the failure and consequent loss of an element. However, as power grids grow in size and get more complex, the number of contingencies increases significantly, increasing the associated computational time. This motivates research towards alternative techniques (Bompard et al. 2009; Gutierrez et al. 2013; Hines et al. 2010a).

Disruptions in networks can be caused by unintentional failures or intentional attacks. Unintentional failures can include manufacturing defects, malfunction in network elements or human error. These kinds of failures can occur randomly throughout the grid and are characterized as random failures (Trajanovski et al. 2013). Intentional attacks or *targeted attacks*, on the other hand, are not random and are aimed at maximizing damage (Rueda et al. 2017). A major challenge in power grids is to evaluate the vulnerability of a power system to these intentional hazards, starting by quantifying the importance of electrical buses and the impact of the attacks on the network performance.

Power grids are amongst the largest and the most complex man-made systems and, like many other complex networks, it features a specific topology which characterizes its connectivity and influences the dynamics of processes executed on the network. The complex nature of power grids and its underlying structure make it possible to analyse power grids relying on network science (Strogatz 2001; Barabási and Albert 1999). The application of network science on power grids has shown the promising potential to capture the interdependencies between components and to understand the collective emergent behaviour of complex power grids (Crucitti et al. 2004; Chassin and Posse 2005).

Topological investigations of power grids have demonstrated that power grids have several components with significant importance compared to the rest of the network (Koç et al. 2014a). These components are crucial for the grid as their removal can significantly disrupt the operation of the power grids. Identifying these critical components in advance can enable power grid operators to improve system robustness by monitoring and protecting these components continuously. (Bompard et al. 2009; Koç et al. 2014a)

Currently, many studies use a complex networks perspective in analysing power system vulnerabilities (Cuadra et al. 2015; Crucitti et al. 2004; Bompard et al. 2008). A significant part of these studies investigates the relationship between the topology and specific performance metrics in the underlying graph of power grids (Crucitti et al. 2004; Negeri et al. 2015; Rosas-Casals et al. 2007). Such studies focus on the basic structural properties of a graph (such as nodal degree, clustering coefficient (Hernández and Van Mieghem 2011)), which typically ignore the electrical properties, such as flow allocation according to Kirchhoff’s laws or the impedance values of transmissions lines in the grid. Mainly, two different aspects are important in the operation and consequent robustness of power grids: the topology of the network formed by electrical buses and their interconnections, and the operating conditions such as supply and demand distributions (Bompard et al. 2012; Cetinay et al. 2016). Consequently, these purely topological metrics could result in misleading research results, which may be far from real physical behaviours of power grids (Arianos et al. 2009; Hines et al. 2010b; Koç et al. 2014a).

To include the electrical properties of the grid in the analyses, several studies propose extended metrics (such as effective graph resistance (Koç et al. 2014b), the electrical centrality (Hines and Blumsack 2008) and the net-ability (Bompard et al. 2012)) by introducing a set of link weights (such as distance or resistance (Qi et al. 2015)) and node properties (such as the electrical demand and supply (Bompard et al. 2012)). Additionally, other studies have used topological and electrical metrics to rank the electrical buses and lines in power grids as a selective contingency analysis (Gorton et al. 2009; Nasiruzzaman et al. 2011).

Motivated by the increasing need of alternative studies to the flow-based analyses and the merits of network science on the investigation of power grids, in this paper, we combine both of the aforementioned approaches: First, we present two different graph representations for a power grid: a simple graph and an extended graph representation that takes the electrical properties of power grids into account. Next, we develop a methodology to identify the critical electrical buses (nodes) in power grids, and compare the impact of targeted node-attacks in detail for European high-voltage transmission networks (Cetinay 2018) and for the publicly available IEEE test power grids (IEEE 2018). Our contributions can be summarized as follows: (i) we consider two different graph models for power grids based on either purely topological information or by including the link weight information and the linearised DC power flow equations; (ii) we employ these two graph models to formulate the standard and the extended centrality metrics of nodes in power grids; (iii) we formulate 8 different attack scenarios according to these centrality metrics and empirically investigate the impact of targeted node-attacks on the structural and operational performance of power grids.

## Power grids and network science

In this section, we provide details about power grids, the steady-state power flow equations and our models for power grids as simple and weighted graphs.

### Power grids preliminaries

Power grids consist of nodes (electrical buses) and interconnecting links (transmission lines and transformers). The status of each node *i* is represented by its voltage $\phantom {\dot {i}\!}v_{i}=|v_{i}| e^{\mathbf {i}\theta _{i}}$ in which |*v*_*i*_| is the voltage magnitude, *θ*_*i*_ is the phase angle, and **i** denotes the imaginary unit. Each line *l* has a predetermined capacity $\mathcal {C}_{l}$ that bounds its flow *f*_*l*_ under a normal operation of the system. In the steady-state of a power grid with *N* nodes and *L* links, the injected apparent power *p*_*i*_+**i***q*_*i*_ at node *i*, where *p*_*i*_ is the active power and *q*_*i*_ is the reactive power, is calculated using the AC power flow equations (Grainger and Stevenson 1994):

1 $$\begin{array}{*{20}l} p_{i}&=\sum\limits_{k=1}^{n} |v_{i}||v_{k}| \left(y^{(\mathrm{R})}_{ik}\cos\theta_{ik} + y^{(\mathrm{I})}_{ik}\sin\theta_{ik}\right)  \end{array} $$

2 $$\begin{array}{*{20}l} q_{i}&=\sum\limits_{k=1}^{n} |v_{i}||v_{k}| \left(y^{(\mathrm{R})}_{ik}\sin\theta_{ik} - y^{(\mathrm{I})}_{ik}\cos\theta_{ik}\right) \end{array} $$

where *θ*_*ik*_=*θ*_*i*_−*θ*_*k*_ and $y^{(\mathrm {R})}_{ik}=\text {Re} (y_{ik})$ and $y^{(\mathrm {I})}_{ik}=\text {Im} (y_{ik})$ are the real and the imaginary parts of the element *y*_*ik*_ in the bus admittance matrix **Y** corresponding to the *i*^th^ row and *k*^th^ column, respectively.

Each node in a power grid contains a number of electrical devices and according to those, two basic types of nodes can be defined (Bergen and Vittal 1999):

- Supply node: A supply node generates the active power *p*_*i*_ and controls the voltage magnitude |*v*_*i*_| at its node *i*.
- Demand node: At a demand node, it is possible to specify the extracted active *p*_*i*_ and the reactive powers *q*_*i*_ from the type of the electrical loads that are connected to that node. There are also nodes without a supply or a demand connected, which can be modelled as a demand node with no injected power, i.e., *p*_*i*_=0 and *q*_*i*_=0.

Due to the impedance of transmission elements, there are power losses during the operation in power grids. As the losses are dependent on the system state–the supply and demand dispatches–they cannot be calculated in advance. Therefore, a slack node among the supply nodes is assigned in power grids to compensate for the difference between the total supply and the total demand plus the losses.

### DC power flow equations

The AC power flow Eqs. (1) and (2) are non-linear and the solution process is generally iterative. A linear set of equations is more desirable whenever fast and repetitive solutions are needed. Linearisation can be reasonably accurate when the following conditions are met (Cetinay et al. 2017; Van Hertem et al. 2006):

1. The difference between the phase angles of neighbouring nodes is small such that sin*θ*_*ik*_≈*θ*_*ik*_ and cos*θ*_*ik*_≈1.
2. The active power losses are negligible, and therefore, the bus admittance matrix can be approximated as **Y**≈**i***Y*^(I)^ where *Y*^(I)^ is the imaginary part of the admittance matrix **Y**, calculated neglecting the line resistances.
3. The variations in the voltage magnitudes |*v*_*i*_| are small and, can be assumed as |*v*_*i*_|=1 for all nodes.

If these conditions are approximately met, the AC power flow equations can be simplified to the so-called the DC power flow equations:

3 $$ p_{i} = \sum\limits_{k=1}^{N} y^{\text{(I)}}_{ik}(\theta_{i}-\theta_{k}).  $$

Although the DC power flow solution is less accurate than the AC power flow solution, in practice, the differences in high-voltage transmission networks between the phase angles of neighbouring buses and the variations in voltage magnitudes are relatively small, thus the error is assumed to be negligible (Van Hertem et al. 2006).

### Graph representations of power grids

This section presents our models for power grids as simple and weighted graphs.

#### Power grid as a simple graph

A simple graph is an unweighted, undirected graph containing no self-loops or multiple links. A power grid can be modelled as a graph $G(\mathcal {N},\mathcal {L})$ where $\mathcal {N}$ denotes the set of *N* nodes and $\mathcal {L}$ denotes the set of *L* links in which multiple lines connecting the same pair of nodes are modelled as one link. The *N*×*N* adjacency matrix **A** specifies the interconnection pattern of the graph $G(\mathcal {N},\mathcal {L})$: *a*_*ik*_=1 only if the pair of nodes *i* and *k* are connected by a direct link; otherwise *a*_*ik*_=0. The *N*×*N* Laplacian matrix **Q** is defined as

$$ \mathbf Q=\mathbf \Delta-\mathbf A $$

where **Δ**=diag(*d*_1_,…,*d*_*N*_) is the diagonal degree matrix with the diagonal elements $d_{i}=\sum _{k=1}^{N} a_{ik}$.

#### Power grid as a weighted graph

Alternatively, a power grid can be modelled as a weighted graph where each link is assigned a weight that is related to the admittance of the transmission line it represents. We model a power grid as a weighted graph ${G(\mathcal {N},\mathcal {L})}$ where $\mathcal {N}$ denotes the set of *N* nodes and $\mathcal {L}$ denotes the set of *L* links^1^. By writing the DC power flow equations in (3) in terms of the adjacency matrix **A** of $G(\mathcal {N},\mathcal {L})$

4 $$\begin{array}{*{20}l} p_{i} &= \sum\limits_{j=1}^{N} a_{ij}y^{\text{(I)}}_{ik}(\theta_{i}-\theta_{j}) = \theta_{i}\sum\limits_{j=1}^{N} {a_{ij}} y^{\text{(I)}}_{ik}-\sum\limits_{j=1}^{N} {a_{ij}} y^{\text{(I)}}_{ik}\theta_{j} \end{array} $$

we introduce the weighted adjacency matrix $\tilde {\mathbf {A}}$, where each nonzero element $\tilde {a}_{ij}={a_{ij}}y^{\text {(I)}}_{ik}$ represents both the connectivity and the admittance between nodes *i* and *j*. Equation (4) can then be written as:

5 $$  p_{i} = \theta_{i}\sum\limits_{j=1}^{N} \tilde{a}_{ij}-\sum\limits_{j=1}^{N} \tilde{a}_{ij}\theta_{j}.  $$

Since (5) holds for every node *i*, the corresponding matrix representation is

6 $$\begin{array}{*{20}l}  \mathbf P & =\left\lbrace \mathbf{{diag}} \left(\sum\limits_{k=1}^{N} \tilde{a}_{ij}\right) - \tilde{\mathbf{A}} \right\rbrace \mathbf{ \Theta}  \\ & = \left(\tilde{\mathbf{\Delta}}-\tilde{\mathbf{A}}\right) \mathbf{ \Theta} \end{array} $$

where **P**= [*p*_1_…*p*_*N*_]^T^ is the vector of net active power injection at the nodes with a balanced supply and demand, i.e. *u*^T^**P**=0 where **u** is all-one vector, $\tilde {\mathbf {\Delta }}$ is the weighted diagonal degree matrix, and **Θ**= [ *θ*_1_…*θ*_*N*_]^T^ is the vector of phase angles at the nodes. Finally, introducing the weighted Laplacian $ \tilde {\mathbf {Q}}= \tilde {\mathbf {\Delta }}-\tilde {\mathbf {A}}$ into (6) yields

7 $$  \mathbf P = \tilde{\mathbf{Q}} \mathbf{ \Theta}  $$

where the weighted Laplacian $\tilde {\mathbf {Q}}$ is a symmetric, positive semi-definite matrix that possesses non-negative eigenvalues apart from the smallest eigenvalue, which is zero (Van Mieghem 2010).

The inversion of the *active power - phase angle* relation $\boldsymbol {P} =\tilde {\mathbf {Q}} \mathbf { \Theta }$ in (7) is not possible due to the fact that det$\tilde {\mathbf {Q}} =0$, which follows from the characteristic property $\tilde {\mathbf {Q}}\mathbf u=0$ of the weighted Laplacian. Although the inverse of the weighted Laplacian matrix does not exist, the *active power - phase angle* relation inversion can be shown to be $\mathbf { \Theta }= \mathbf {Q^{\dag }} \mathbf {P}+\frac {\mathbf {u^{\mathrm {T}}} \mathbf {\Theta } }{N} \mathbf u $, where *Q*^*†*^ is the pseudo-inverse of the weighted Laplacian $\tilde {\mathbf {Q}}$, obeying $\tilde {\mathbf {Q}}\mathbf {Q^{\dag }}=\mathbf {Q^{\dag }} \tilde {\mathbf {Q}}=\mathbf {I}-\frac {1}{N}\mathbf {J}$ with the identity **I** and all-one matrix ***J***=**u***u*^T^. By choosing the average phase angle in the graph $ {\theta _{\text {av}}}=\frac {\mathbf {u}^{\mathrm {T}} \mathbf {\Theta }}{N} =0$ as the reference (Cetinay et al. 2016), the *phase angle - active power* relation takes the elegant form of

8 $$ \mathbf{\Theta}=\mathbf{Q^{\dag}}\mathbf{P}.   $$

While the weighted Laplacian $\tilde {\mathbf {Q}}$ and its pseudo-inverse *Q*^*†*^ are derived here based on the linearised DC power flow equations in power grids, their applicability is far wider (Van Mieghem et al. 2017). A weighted Laplacian $\tilde {\mathbf {Q}}$ can describe many processes, that are linear in or proportional to the network topology such as electrical circuits, water flow networks, mechanical or thermal systems. The process equivalence between those systems are given in Table 1.

**Table 1:** Equivalence between linear systems, adopted from (Van Mieghem et al. 2017)

Power grids	Phase angle	Power
Electrical circuit	Voltage	Current
Hydraulic circuit	Pressure (height of liquid)	Volume flow
Mechanical system	Force	Displacement velocity
Thermal system	Temperature	Heat flow
…	…	…

## Targeted attacks on power grids

The threats for power grids can be classified by using multiple criteria considering the causes of the threat, their consequences or the preventive actions to manage the hazards (Ciapessoni et al. 2017). One example of such threats are targeted attacks on power grids, which involve intentional, criminal actions to destroy the network. In modelling these threats, we assume that the attacks are performed with the knowledge of power grid layout and with the intention to maximally disrupt the network performance while attacking as few nodes (electrical buses) as possible. Throughout this section, we describe how network science can be employed to formulate such attack strategies, where target nodes correspond to most critical or most vulnerable nodes whose removal significantly disrupts the network functioning. We first describe the standard centrality metrics, which are purely based on the underlying topology of power grids, and then we extend these metrics to include the information on the link weights, i.e. the admittances of the transmission lines, and the DC power flow equations in power grids.

### Ranking nodes in the simple graph representation of a power grid

In this section, we review some of the existing topological centrality metrics in order to rank the importance and the centrality of nodes in the underlying simple graph of power grids.

#### Degree centrality

The degree *d*_*i*_ of a node *i* in the graph $ G(\mathcal {N},\mathcal {L})$ is equal to the number of its neighbouring nodes (Freeman 1978). The degree *d*_*i*_ can be calculated using the adjacency matrix **A**:

9 $$  d_{i}=\sum_{j=1}^{N} a_{ij}.  $$

#### Eigenvector centrality

The eigenvector centrality of a node is a global centrality metric that depends not only on the number of its neighbouring nodes, but also on the number of 2-hop neighbouring nodes, 3-hop neighbouring nodes, and so on (Bonacich 1987; 1991). The eigenvector centrality *x*_*i*_ of node *i* is equal to the *i*^th^ component of the eigenvector corresponding to the largest eigenvalue *λ*_1_ of the adjacency matrix **A**. The principal eigenvector centralities thus follow from the linear equations:

10 $$  x_{i} = \frac{1}{\lambda_{1}} \sum_{k=1}^{N} a_{ik} x_{k}.  $$

#### Betweenness centrality

Another metric to assess node importance or centrality is the betweenness centrality (Freeman 1977). blackIn calculating the betweenness centrality, it is assumed that information or services are transmitted over shortest paths between node pairs. Hence, if many shortest paths pass through a certain node, this node takes a central role in the network. If $\vert \mathcal {P}_{s\rightarrow t}\vert $ is the number of all possible shortest paths from node *s* to node *t*, and $\vert \mathcal {P}_{s\rightarrow t}(i)\vert $ is the number of those paths that pass through node *i*, then the betweenness *b*_*i*_ of node *i* is equal to

11 $$  b_{i}=\sum_{s,t\in \mathcal{N} \setminus \{i\}} \frac{\vert\mathcal{P}_{s\rightarrow t}(i) \vert}{\vert\mathcal{P}_{s\rightarrow t}\vert}.  $$

In other words, the betweenness centrality of a node *i* shows the fraction of all shortest paths between any pair (*s*,*t*) of nodes, that pass through node *i*.

#### Closeness centrality

In calculating the closeness centrality, the hopcount $H(\mathcal {P}_{i\rightarrow j})$ that is the number of links in the shortest path $\mathcal {P}_{i\rightarrow j} $ between a pair of nodes *i* and *j*, is used. The closeness centrality *c*_*i*_ of a node *i* is defined as (Freeman 1977):

12 $$  c_{i}= \frac {1} { \sum_{j\neq i} H(\mathcal{P}_{i \rightarrow j }) },  $$

which is the reciprocal of the sum of the hopcounts of node *i* to all other nodes. A large closeness centrality value thus corresponds to a “central” node that is well-connected by a few hops to other nodes.

### Ranking nodes in the weighted graph representation of a power grid

While the standard centrality metrics are based on purely topological information, it is possible to extend the definition of these metrics by including the link weight information and the power flow equations in power grids. Different definitions of extended centrality metrics (extended betweenness (Bompard et al. 2010), modified betweenness and closeness centrality (Gutierrez et al. 2013), electical degree (Hines and Blumsack 2008)) exist^2^ and are evaluated by simulations via power flow solvers or by calculating power transfer distribution factors (PTDF) in power grids. Such simulation-based definitions are generally computationally expensive and formulations with the absence of slack node(s) may not fully explain the analogy between the extended centrality definitions and the weighted graph model for power grids.

Extended metrics were also defined before (Ellens et al. 2011; Newman 2005) based on the *voltage - current* relation in electrical circuits. Since the *phase angle - active power* relations in (7) and (8) in power grids obey the same linear relation as those in electrical circuits (as described before in Table 1), these metrics can identify central nodes in power grids.

We take here a graph theoretical approach using the slack-node-independent weighted graph representation for power grids described in the previous section. This weighted graph model facilitates both the analogy between the standard and the extended centrality metrics, and the enhanced linear algebra to formulate the closed-form expression of centrality metrics via graph-related matrices.

#### Weighted degree centrality

Similar to the topological definition in (9), the weighted degree centrality is related to the number of neighbours of a node. However, rather than only considering the number of neighbours, the weighted degree $\tilde {d}_{i}$ also includes the information of the admittances $\tilde {a}_{ij}$ of the transmission lines that link the nodes, which leads to the definition:

13 $$  \tilde{d}_{i}=\sum\limits_{j=1}^{N} \tilde{a}_{ij}.  $$

A large value of the weighted degree $\tilde {d}_{i}$ corresponds to larger values of the admittance directly connected to that node, which indicate that node *i* is well connected to its neighbours.

#### Weighted eigenvector centrality

In analogy with the eigenvector centrality in (10), the weighted eigenvector centrality $\tilde {x}_{i}$ not only captures the total admittance of all lines connected to node *i*, but is also influenced by the admittance of all lines connected to its neighbours, their neighbours and so on. The weighted eigenvector centralities correspond to the eigenvector of the highest eigenvalue $\tilde {\lambda }_{1}$ of the weighted adjacency matrix $\tilde {\mathbf {A}}$. Thus, the principal weighted eigenvector centrality $\tilde {x}_{i}$ is given by the equation:

14 $$  \tilde{x}_{i} = \frac{1}{\tilde{\lambda}_{1}} \sum_{j=1}^{N} \tilde{a}_{ij} \tilde{x}_{j}.  $$

#### Flow betweenness centrality

While in the standard definition of the betweenness and the closeness centrality in (11) and (12), information exchange and other processes are assumed to travel over shortest paths, in the case of the DC power flow equations (or in the equivalent linear systems in Table 1), the flow distribution obeys Kirchhoff’s and Ohm’s laws. Therefore, the standard betweenness and closeness centrality based on shortest paths may not fully capture the operation of power grids. Instead, the flow betweenness centrality $\tilde {b}_{i}$ of node *i* depends on the total flow running through that node, as proposed by Newman (2005):

15 $$ \tilde{b}_{i} = \sum\limits_{s,t\in \mathcal{N} \setminus \{i\}} \sum\limits_{j \in\mathcal{B}(i)} \vert f_{s\rightarrow t}(i,j)\vert,  $$

where $\mathcal {B}(i)$ denotes the direct neighbours of node *i*, and |*f*_*s*→*t*_(*i*,*j*)| is the magnitude of the power flow through the link between *i* and *j* when a unit active power is injected at node *s* and extracted from node *t*. In Appendix 2, we show how these flows can be calculated from the weighted graph representation of a power grid. Higher values of the flow betweenness centrality $ \tilde {b}_{i}$ indicate the importance of a node with respect to the electrical power transmission in power grids.

#### Electrical closeness centrality

Similar to the definition of the closeness centrality in (12), the electrical closeness centrality of a node is an indicator of the average distance of that node to all other nodes. However, since the flow in a power grids obeys Kirchhoff’s laws, the effective resistance (Ellens et al. 2011; Cetinay et al. 2016) is a more appropriate distance metric between nodes than the hopcount of shortest-path. The effective resistance *Ω*_*ij*_ between a pair of nodes can be calculated from the pseudo-inverse Laplacian matrix as (Van Mieghem 2010):

$$\Omega_{ij} = (\mathbf{Q}^{\dagger})_{ii} + (\mathbf{Q}^{\dagger})_{jj} - 2(\mathbf{Q}^{\dagger})_{ij}, $$

and captures the effect of the active power transfer *p*_*ij*_ and the phase angle difference *θ*_*i*_−*θ*_*j*_ between a pair of nodes, when active power is only injected at and extracted from nodes *i* or *j*:

$$\Omega_{ij} = \frac{\theta_{i}-\theta_{j}}{p_{ij}}. $$

Since the effective resistance satisfies the properties^3^ of a distance function (Klein and Randić 1993) and obeys the flow equations in power grids, it can be used to define a distance-based centrality metric. The electrical closeness centrality $\tilde {c}_{i}$ of a node equals the reciprocal of the total effective resistance of that node to all other nodes^4^:

16 $$ \tilde{c}_{i} = \frac{1}{\sum_{j=1}^{N}\Omega_{ij}}.  $$

Compared to the shortest-path hopcount $H(\mathcal {P}_{i\rightarrow j})$, the effective resistance *Ω*_*ij*_ does not depend only on the shortest path, but also incorporates the information of all possible paths between node *i* and *j*, where the contribution of each possible path follows from the linear flow equations. In the case of the unweighted tree networks, the effective resistance *Ω*_*ij*_ equals the hopcount $H(\mathcal {P}_{i\rightarrow j})$ for all nodes. Thus, for tree-like power grids with equal admittances, the electrical closeness centrality closely resembles the topological closeness centrality, while for power grids with many loops (i.e. non-tree-like) both metrics could differ significantly.

Each of the centrality metrics we present captures a certain aspect of the structural and the operational centrality in the network, such as the strength of a direct connectivity (degree and eigenvector centrality), being a part of many important paths (betweenness centrality) or being close to other nodes (closeness centrality). In recent years, another conceptual definition of centrality has emerged. Based on optimal percolation theory (Morone and Makse 2015), which considers the problem of “finding the smallest set of nodes whose removal fragments the network in small disconnected pieces”, a number of new metrics have been proposed (such as the collective influence (CI) (Morone and Makse 2015; Morone et al. 2016), belief propagation decimation (BPD) (Mugisha and Zhou 2016) and CoreHD (Zdeborová et al. 2016)). Such metrics reflect the importance of a node for the global structural coherence as well as their influence in spreading behaviour. However, to the best of our knowledge, *extended* metrics based on percolation theory have not been studied yet. Therefore, in this work, we focus on the generally accepted and adopted centrality metrics to the power grids.

## Identifying the effect of node removals in power grids

In this section, we empirically compare the effects of the targeted node removals based on the centrality metrics presented in the previous section. To evaluate the change in the network functioning, we use two performance metrics that can quantify both the topological and the operational characteristics of the grid after targeted attacks. We consider the networks from 5 real-world power grids of European countries (Cetinay 2018) and 5 synthetic power grids from the IEEE test case database (IEEE 2018).

### Performance metrics

In an ideal power grid which is robust to targeted attacks, the removal of nodes should not significantly alter the network functioning. In some cases, removing a node from the power grid can partition the network into several components, which are disconnected from each other. This is undesirable as this partition both adversely affects (i) the structure: as the size (i.e. the number of nodes) of the connected component of the network is decreased, and (ii) the operation: since the disconnected structure disrupts the service and the capacity of the network. In this work, we present two performance metrics in our case studies, the size and the capacity of the giant component, to assess both the topological and the operational performance aspect in the network.

#### The size of the giant component

The giant component (Molloy and Reed 1998) is the connected component of a graph that contains the largest fraction of the entire graph’s nodes. The size of the giant component in the graph reflects the disruptive effect of node removals on the structure of the network.

We assume that the underlying graph of the initial network is connected, thus the initial size of the giant component is *N*. Then, we calculate the normalized size *σ* of the giant component after each node removal as the ratio between the size of the current giant component and the initial network size *N*, in other words

17 $$  \sigma = \frac{\sum_{i=1}^{N} 1_{\{i \in G^{\prime}\}}}{N}  $$

where 1_{*x*}_ is the indicator function: 1_{*x*}_=1 if the condition {*x*} is true, else 1_{*x*}_=0, and *G*^′^ is the current giant component of the initial graph *G*(*N*,*L*).

#### The capacity of the giant component

Each transmission line in a power grid is associated with a maximum flow carrying capability. For the safe operation of a network, the flows through the network links should be below these capability. If the flow limits are exceeded, the situation is detected by protection relays, the circuit breakers are tripped, and the corresponding element is taken out of service. The possibility and the negative impact of cascading failures in power grids increases when the operating point of a power grid is close to the flow carrying capabilities of its links (Koç et al. 2013; Cetinay et al. 2017). Consequently, a network with a high flow carrying capability is desired.

We calculate the total capacity of the network as the sum of the maximum flow carrying capabilities of links in the largest connected component of the graph. When multiple lines are connecting the same pair of nodes, we consider an equivalent capacity between those nodes. This equivalent capacity represents the maximum power that can be transferred between these nodes such that the resulting power flow through each single line is at most at its capacity. In Appendix 3, we describe how this equivalent capacity is calculated.

The capacity of the giant component depends on the number of links in the giant component as well as the flow carrying capability of links, which are closely related to the electrical demands and supplies at the neighbouring nodes^5^. We calculate the normalized capacity of the giant component *γ* after each node removal as the ratio between the total capacity of the current giant component and the total capacity of the initial network, in other words

18 $$  \gamma = \frac{\sum_{l=1}^{L} \left(\mathcal{C}_{l} \times 1_{\{l \in G^{\prime}\}}\right)}{\sum_{l=1}^{L} \mathcal{C}_{l}}.  $$

### Properties of the networks used in simulations

**Fig. 1:**
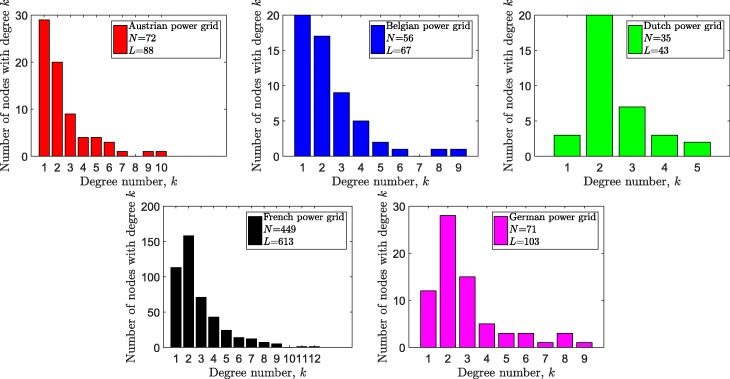
The degree distribution of the simple graphs of 5 European power grids

**Fig. 2:**
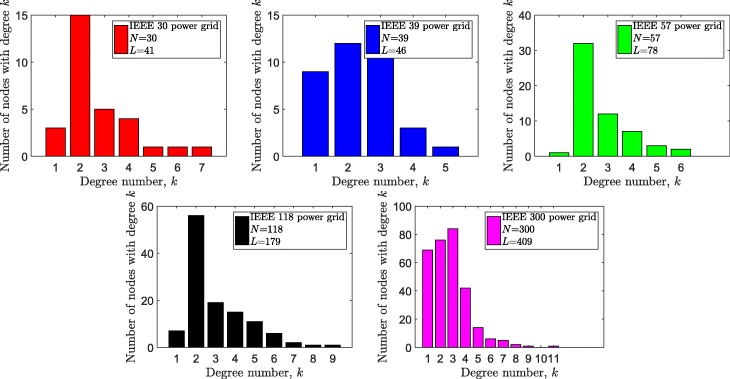
The degree distribution of the simple graphs of 5 IEEE power grids

We considered the high-voltage transmission networks of 5 European countries in our case studies: Austrian, Belgian, Dutch, French and German power grids. In addition, we included 5 widely used test power grids from IEEE database (IEEE 2018). In all networks, multiple lines connecting the same pair of nodes are represented as an equivalent single link using the equivalent admittance (23) and the equivalent maximum flow carrying capability (25). The degree distributions of the underlying graphs are shown Figs. 1 and 2. Additionally, more details of the power grids in our case study are available on our GitHub page (Cetinay 2018).

### The effects of targeted node removals in power grids

We apply both the standard and the extended centrality metrics as node-attack strategies in power grids. For each centrality metric, we start the attacks by removing the node (and all its links) with the highest value of the chosen centrality metric. After each node removal, we recalculate the values of the centrality metric, and continue by removing the node with the highest value of the centrality metric in the current giant component of the graph. Note that, during the successive node removals, we do not take the cascading dynamics (such as overloading of links or demand/supply redistribution due to cascading failures (Cetinay et al. 2017)) into account. In other words, we focus on the instant just after the removal of nodes to identify the effects on the structure and the operational performance indicators of the power grid.

**Fig. 3:**
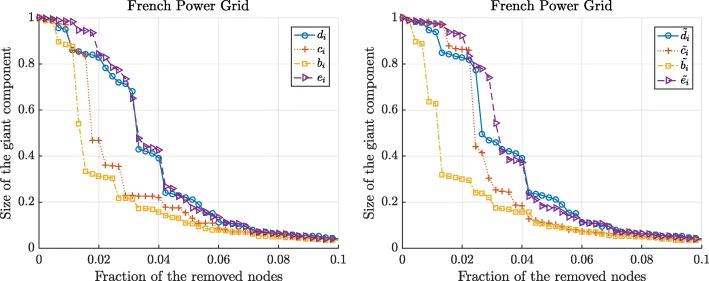
The normalized size of the giant component in the French power grid versus the removal of nodes according to the standard centrality metrics (left) and the extended centrality metrics (right). The node with optimal graph metric, computed in resulting giant component, is removed

**Fig. 4:**
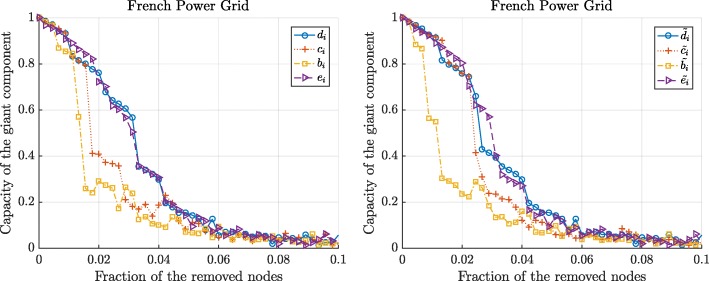
The normalized capacity of the giant components in the French power grid versus the removal of nodes according to the standard centrality metrics (left) and the extended centrality metrics (right). The node with optimal graph metric, computed in resulting giant component, is removed

Figures 3 and 4 show the changes in the normalized size and the capacity of the giant component when we sequentially remove the nodes according to 8 different centrality metrics. We observe that the betweenness and the flow betweenness centrality are the best attack strategies as they can maximally disrupt the network functioning with fewer attacked nodes. On the other hand, the degree centrality may not always successfully assign an important node. Compared to the degree centrality, the betweenness and the flow betweenness centrality give more fine-grained centrality values for each node, whereas, multiple nodes with the same degree exist, as illustrated in Figs. 1 and 2, making them indistinguishable. In addition, we observe that the eigenvector and the weighted eigenvector centrality are the least effective attack strategies: Targeted attacks according to these centrality metrics destroy the network slower than other attack strategies.

Next, in order to compare the attack strategies and to further quantify the topological and operational changes in power grids, we calculate the average of the structural and the operational performance indicators (or the energy *ε* values of a graph (Trajanovski et al. 2013)) that are the normalized sums of the size and the capacity of the giant component over successive targeted attacks, respectively. Thus, the average value $\bar {\sigma } $ of the structural performance indicator of the power grid over *K* successive node-attacks can be calculated as

19 $$  \bar{\sigma} = \frac{\sum_{k=1}^{K} \sigma(k)}{K}  $$

where *σ*(*k*) is the normalized size of the giant component after *k* successive attacks. Similarly, the average value $\bar { \gamma }$ of the structural performance indicator of the power grid over *K* successive node-attacks is

20 $$  \bar{ \gamma }= \frac{\sum_{k=1}^{K} \gamma (k)}{K}  $$

where *γ*(*k*) is the normalized capacity of the giant component after *k* successive attacks. The structural $\bar {\sigma } $ and the operational $\bar { \gamma } $ performance indicators in (19) and (20) are evaluated on a score between 0 and 1: In an ideal power grid which is robust to targeted attacks, the node removals should have slight effects on the network performance. Thus, a performance indicator close to 1 is desirable by the network operators. On the other hand, a lower performance indicator over successive node-attacks indicates a powerful (destructive) attack-strategy in which few important nodes of the network are identified and removed, with negative operational and structural consequences.

**Fig. 5:**
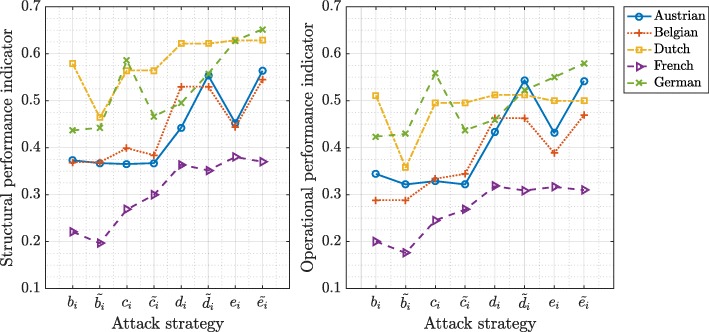
The structural performance indicator $\bar {\sigma }$ (left) and the operational performance indicator $\bar {\gamma }$ (right) in European power grids after the targeted attacks

**Fig. 6:**
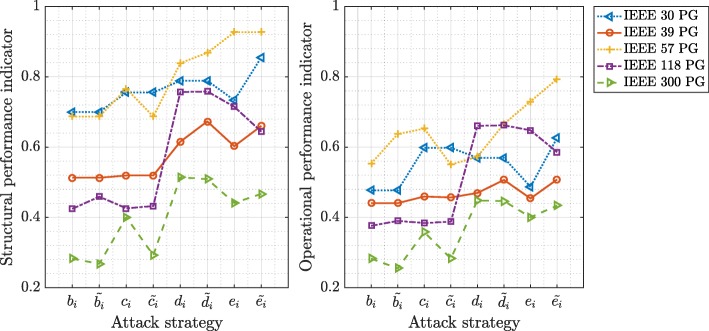
The structural performance indicator $\bar {\sigma }$ (left) and the operational performance indicator $\bar {\gamma }$ (right) in IEEE power grids after the targeted attacks

In Figs. 5 and 6, we present the average values of the performance indicators in European and IEEE test power grids (PG) after different attack strategies that remove 10% of the initial network nodes, respectively. Higher values in Figs. 5 and 6 represent higher robustness to the targeted attacks, whereas lower values indicate vulnerability or a severe disrupt in network functioning. We observe that targeted attacks based on the flow betweenness and the betweenness centrality followed by the closeness and the electrical closeness centrality are the best attack strategies to decrease the structural and the operational performances of the power grids. As an example, the targeted node attacks according to the flow betweenness centrality of nodes destroy the Dutch power grids faster than any other attack strategy.

### Main lessons learned from the analyses

In this section, we summarize the insights obtained in the previous sections. The main lessons learned from the analyses of the targeted node-attacks based on the different centrality metrics from the simple and the weighted graph representations of power grids in the tested networks are as follows:

- The degree centrality (9) only provides information on the local structure around a node. Similarly, the weighted degree centrality (13) reflects local connectivity information. Thus, a node that is connected to many other nodes (with high admittance) is not necessarily a central node for the whole network. Therefore, as illustrated by the targeted attack simulations, the degree and the weighted degree centralities cannot always indicate the important nodes.
- The betweenness centrality (11) incorporates information about the global network structure, and in the analyses of the test networks, high betweenness centrality values were found to efficiently indicate the nodes whose removal would significantly disrupt the network performance. While successfully indicating vulnerable nodes, the betweenness centrality (11) is based on the shortest paths only. This means that the betweenness centrality does not discriminate nodes that are positioned “close” to many shortest paths (and would be considered central), and peripheral nodes. This limitation is partly addressed by the flow betweenness centrality (15), in which the flows through the network links are distributed throughout the network according to the Kirchhoff’s laws. In the analyses of the test networks, removing nodes with a high flow betweenness usually resulted in the most destructive effects on the network.
- The closeness centrality (12) reflects the average shortest path distance from a node to all other nodes in the network. Higher closeness centrality values thus indicate nodes which can easily reach the other nodes in the network. Similarly, higher values of electrical closeness centrality (16) show a node that is on average close to the other nodes in the network, based on the operationally inspired effective resistance distance instead of the shortest-path distance. In the analyses of targeted attacks, the performance of the closeness and the electrical closeness centrality in identifying the important nodes in the tested power grids are found to be similar.
- The eigenvector centrality (10) can rarely identify the critical nodes, and thus, the targeted attacks based on the eigenvector centrality are generally the worst destructive strategy among the traditional centrality metrics in the tested networks. Similarly, the weighted eigenvector centrality (14) seems not to successfully indicate important nodes.

The analyses of the targeted node-attacks show that centrality metrics, in particular the (flow) betweenness and (electrical) closeness, are very successful in indicating the critical nodes whose removals sharply decrease the selected performance indicators (the size and the capacity of the giant component) of power grids. Identifying these critical components in advance can enable power grid operators to improve system robustness by monitoring and protecting these components continuously. Additionally, although the effect of targeted attacks are more significant when the centrality information is updated after each node removal, the information based on the initial calculation of the centrality metrics is also fairly successful in finding the important nodes. The degree centrality is a good indicator to fragment the network to decrease the structural and operational performance indicators of power grids (See Appendix 1).

## Conclusion

In this paper, we took a network science approach to investigate the vulnerability of power grids to malicious targeted attacks. First, we presented two different graph models for power grids: simple and weighted graphs. Subsequently, using these graph models, we ranked the importance of each node according to the standard and the extended centrality metrics that take into account the electrical properties of the grids such as the admittance of the transmission lines and the flow allocation according to the DC power flow equations. Via case studies in both real-world and test power grids, we show that the power grids are highly vulnerable to targeted attacks: sequentially removing the nodes with the highest centrality is a good strategy to fragment the power grids, and to maximally decrease its operational performance. In almost all power grids in our case study, removing approximately 15% of the nodes according to the flow betweenness centrality destroys the network almost completely. Grid operators can use the proposed methodology to analyse the current vulnerability of their network to targeted attacks and to take necessary measures by protecting the important nodes in their networks.

## Appendix 1: Targeted attacks based on initial centrality metrics

Instead of recalculating the centrality metrics after each node removal, we consider here a more simplified attack strategy based on calculating the centrality metrics only once, at the beginning of the attacks. The targeted attacks are then performed sequentially according to these initial values.

**Fig. 7:**
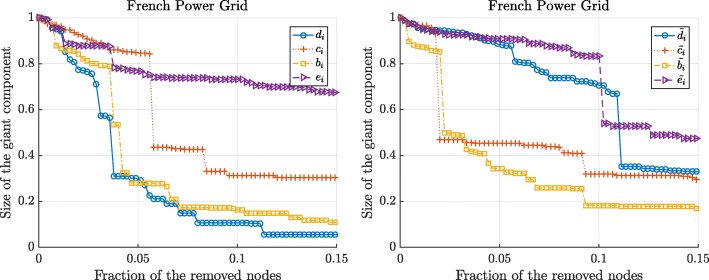
The normalized size of the giant component in the French power grid versus the removal of nodes according to the standard centrality metrics (left) and the extended centrality metrics (right). Nodes are removed sequentially according to the initial values of the centrality metrics

**Fig. 8:**
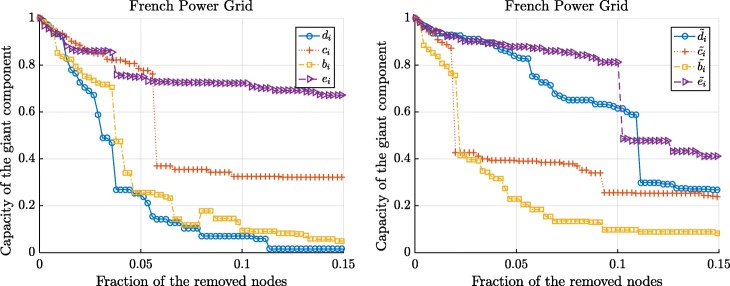
The normalized capacity of the giant component in the French power grids versus the removal of nodes according to the standard centrality metrics (left) and the extended centrality metrics (right). Nodes are removed sequentially according to the initial values of the centrality metrics

Figures 7 and 8 show the changes in the normalized size and the capacity of the giant component in French power grids after the targeted attacks, respectively. Figures 7 and 8 illustrates that even these simplified attack strategies could inflict a significant damage on the network functioning: For instance, removal of 15% of the nodes according to the initial rankings nearly destroys French power grids. Compared to Figs. 3 and 4, in Figs. 7 and 8, we observe that the degree centrality is the most destructive attack strategy when the centrality metrics are based on only the initial calculation of the centrality metrics, i.e. when the node-rankings are not updated after the targeted attacks.

## Appendix 2: Calculation of flow betweenness centrality in power grids

Following the linearised DC power flow equations in power grids, the active power **P** and phase angle **Θ** in all nodes are related by Eqs. (7) and inversely by (8). When a unit active power is injected at node *s* and extracted at node *t*, this corresponds to the active power input to the grid:

$$\mathbf{P}_{s\rightarrow t} = \mathbf{e}_{s}-\mathbf{e}_{t} $$

where **e**_*k*_ is the basis vector with the *k*^th^ component equal to one and all other components zero. Based on Eq. (8), the resulting phase angle vector for this active power input can be calculated as

$$\mathbf{\Theta}_{s\rightarrow t} = \mathbf{Q}^{\dagger}(\mathbf{e}_{s}-\mathbf{e}_{t}). $$

Knowing the phase angle at each node, it is then possible to calculate the flow *f*_*s*→*t*_(*i*,*j*) through the link between nodes *i* and *j* as

21 $$ f_{s\rightarrow t}(i,j) = \tilde{a}_{ij} (\mathbf{e}_{i}-\mathbf{e}_{j})^{\mathrm{T} }\mathbf{\Theta}_{s\rightarrow t}.  $$

The flow betweenness centrality $\tilde {b}_{i}$ of a node *i* is the sum of the absolute flows that pass through that node *i*, over all possible pairs of source and target nodes^6^:

22 $$ \tilde{b}_{i} = \sum_{s,t\in \mathcal{N} \setminus \{i\}} \sum_{j \in\mathcal{B}(i)} \left\vert \tilde{a}_{ij} (\mathbf{e}_{i}-\mathbf{e}_{j})^{\mathrm{T}} \mathbf{Q}^{\dagger}(\mathbf{e}_{s}-\mathbf{e}_{t})\right\vert.  $$

## Appendix 3: Multiple lines connecting the same pair of nodes

We consider multiple lines $\mathcal {L}^{\prime }$ connecting the same pair of nodes *i* and *j*: each line *l* has admittance *y*_*l*_ and flow capacity $\mathcal {C}_{l}$. In the weighted graph model for power grids, those multiple lines $\mathcal {L}^{\prime }$ are represented as a single equivalent link between node *i* and *j*, with admittance

23 $$ y^{\text{(I)}}_{ij} = \sum_{l \in \mathcal{L}^{\prime}} y^{\text{(I)}}_{l}.  $$

The maximum possible flow between those nodes *i* and *j* is constrained by the capacity of each single line connecting them. If power *f*_*ij*_ flows from node *i* to node *j*, then according to the DC power flow equations in (3), this results in the phase angle difference

24 $$ (\theta_{i}-\theta_{j}) = \frac{f_{ij}}{y_{ij}^{\text{(I)}}},  $$

where $y_{ij}^{\text {(I)}}=\sum _{l \in \mathcal {L}^{\prime }} y_{l}^{\text {(I)}}$ is the equivalent admittance between node *i* and *j* of $\mathcal {L}^{\prime }$ lines in parallel. For each single line, Ohm’s law states that the flow *f*_*l*_ through that line is related to the phase angle difference by

$$f_{l} = y_{l}^{\text{(I)}}(\theta_{i}-\theta_{j}). $$

Introducing the phase angle difference from Eq. (24) then leads to

$$f_{l} = f_{ij}\frac{y_{l}^{\text{(I)}}}{y_{ij}^{\text{(I)}}} $$

for the flow *f*_*l*_ through line *l*. Since the maximum flow through each line is constrained by its flow capacity: $f_{l}\leq \mathcal {C}_{l}$, we find that the total flow *f*_*ij*_ between node *i* and *j* is constrained by an equivalent capacity $\mathcal {C}_{ij}$ equal to:

$$f_{ij}\leq \mathcal{C}_{ij} $$

where,

25 $$ \mathcal{C}_{ij} = \underset{l \in \mathcal{L}^{\prime} }{\min}\left(\frac{\mathcal{C}_{l}}{y_{l}^{\text{(I)}}}\right)y_{ij}^{\text{(I)}}.  $$

## Footnotes

1 Multiple lines connecting the same pair of nodes are represented as a single equivalent link in the graph, see Appendix 3.

2 A broad review of robustness studies in power grids using network science can be found in (Cuadra et al. 2015).

3 The properties of a distance function *D*(*i*,*j*) between a pair of nodes *i* and *j* are: (a) non-negativity: *D*(*i*,*j*)≥0, (b) zero distance for identical nodes: *D*(*i*,*j*)=0 if and only if *i*=*j*, (c) symmetry: *D*(*i*,*j*)=*D*(*j*,*i*) and (d) the triangle inequality: *D*(*i*,*k*)+*D*(*k*,*j*)≥*D*(*i*,*j*). The effective resistance *D*(*i*,*j*)=*Ω*_*ij*_ satisfies all four properties (Klein and Randić 1993).

4 Van Mieghem et al. (2017) defines the node *i*^⋆^ which is electrically the best spreader to all other nodes based on the flow equations in electrical circuits. This best spreader node *i*^⋆^ corresponds here to the node with the highest electrical closeness centrality, i.e. $i^{\star } = \underset {i \in \mathcal {N}}{\operatorname {argmax~}}\tilde {c}_{i}$.

5 Another metric that can be used to capture the operational performance of power grids is the *Yield* which is the ratio of the total demand supplied at the end of an attack with respect to the initial demand of the network (Cetinay et al. 2017). This electrical demand information, which is needed to calculate Yield, is not available in our data sets of real-world power grids.

6 In power grids, the flow through network links are directed. Different from load transmissions in other types of networks, opposite directed flows through a link can cancel out the total flow through that link. Therefore, to calculate the maximum possible flow through a link, the absolute sum is necessary. This definition under the maximum possible loading condition is also used in the definition of *full betweenness centrality* by (Martín Hernández et al. 2014).
